# Psychological Resilience in Young Female Athletes

**DOI:** 10.3390/ijerph18168668

**Published:** 2021-08-17

**Authors:** Kimberly H. McManama O’Brien, Miriam Rowan, Kyra Willoughby, Kelsey Griffith, Melissa A. Christino

**Affiliations:** 1 Division of Sports Medicine, Department of Orthopedic Surgery, Department of Psychiatry, Boston Children’s Hospital, Harvard Medical School, Boston, MA 02115, USA; kimberlyh.m.obrien@childrens.harvard.edu (K.H.M.O.); miriam.rowan@childrens.harvard.edu (M.R.); 2 Harvard University, Cambridge, MA 02138, USA; kyrawilloughby@college.harvard.edu; 3 The Micheli Center for Sports Injury Prevention, Division of Sports Medicine, Department of Orthopedic Surgery, Boston Children’s Hospital, Boston, MA 02115, USA; kelsey.griffith@childrens.harvard.edu; 4 Division of Sports Medicine, Department of Orthopedic Surgery, Boston Children’s Hospital, Harvard Medical School, Boston, MA 02115, USA

## Abstract

Psychological resilience is an important construct that can enhance athletic performance and foster valuable life skills. Through positive adaptation to adversity and stressors in the athletic arena, athletes are able to cultivate their ability to effectively respond to negative stimuli, ultimately evolving to personal growth. For young female athletes, development of resilience may be particularly important. Young female athletes face distinct challenges in sport including sport inequity, body image issues, eating disorders, increased mental distress, and internalization of emotions. The aim of this review is to define and describe the construct of resilience and discuss the implications and applications relevant to young female athletes. By understanding how to foster resilience strategies in this population, we can enhance sport performance and enjoyment, as well as bolster valuable life skills that facilitate personal growth.

## 1. Introduction

Girls’ participation in sports has steadily grown since the 1970s [1], driving the need to better understand the psychology of female athletes [2]. Athletic participation yields many benefits. From a psychological standpoint, sport participation can positively impact self-esteem and mood [3,4,5]. In addition to psychological benefits, sport participation also improves physical health [6] and has been associated with decreased substance use [7]. While sport clearly has many benefits for girls, athletic participation may simultaneously present compounding psychosocial stressors, daily hassles, and adverse events in the height of a sensitive [8,9,10] developmental period [11].

Developing resilience (i.e., experiencing adversity and having a positive adaptation to that adversity) is a necessary step in an athlete acquiring high levels of performance [12]. Without positive adaptation to adversity in the athletic environment, young female athletes may experience unwanted developmental consequences, such as poor coach relationships, negative peer influences, parent pressure, and the challenging psychological environment of competitive sport [13,14].

Given the unique opportunities that sport presents, with respect to goal achievement and overcoming adversity on a frequent basis, it is critical that protective factors be identified, understood, and fostered in young female athletes. Such an endeavor requires examination of the dynamic construct of psychological resilience which can positively affect an athlete’s performance and well-being [15,16,17]. The goal of this review is to define psychological resilience, explain how it benefits young female athletes, and discuss ways to enhance this skill in order to build capacity for optimal performance and enjoyment in sport, as well as bolster valuable life skills that facilitate personal growth.

## 2. Defining Psychological Resilience

The American Psychological Association defines psychological resilience as “the process of adapting well in the face of adversity, trauma, tragedy, threats, or significant sources of stress” [18]. In a recent review of the concept of psychological resilience by a multidisciplinary panel of experts, psychological resilience was defined as “an effective adaptation to, or a navigation (or management) of, significant sources of traumatic stress or adversity and the capacity to absorb disturbance to harness resources effectively” [16]. More generally, researchers have defined resilience as an individual’s “stress coping ability” [19], as well as the capacity for “bouncing back” from adversity [20]. Other constructs considered alongside resilience in athletic research include “well-being” and “thriving” in the presence of stressors and hassles of everyday life [21]. For young female athletes to be resilient, they require the development of the interconnected core skills of positive adaptation to diversity [21] and the ability to cope with stress [19,22]. In sum, developing psychological resilience in sport is a dynamic process that requires positively adapting to stress.

## 3. Distinct Issues Facing Young Female Athletes that Require Resilience

Female athletes have particular experiences and vulnerabilities at odds with the development of resilience. Lunde and Gattario (2017) note that “young women who engage in sport have to face complex, ambiguous, and restricting norms and notions” that may impact their health and performance [23].

### 3.1. Gender Inequality and Discrimination

Female athletes of all ages face issues of gender inequality and discrimination. Historically, athletic opportunities have favored boys over girls. Since the advent of Title IX in 1972, opportunities and funding for women and girls to pursue athletic endeavors and participate in organized sport have expanded [24]. Despite this progress, what has emerged is the sexualization, devaluation, and feminist-trait stereotyping of elite female athletes by sport media, including image and print [14,25,26,27,28], and the female youth who idolize, admire, and mimic these female athletes are exposed to such messages with some impact. For example, when female youth athletes were shown sexualized images of female athletes, these youth made significantly greater self-objectifying statements [29], indicating they were experiencing themselves as if from a third-person perspective [30]. This self-objectification can have effects on sport performance; one study demonstrated that higher levels of self-objectifying tendencies were associated with poorer softball throwing in female youth athletes aged 10 to 17 years old [31].

### 3.2. Body Image

One distinct concern for female athletes is highlighted in research on contextual body image [32,33], whereby it is understood that some athletes may aspire towards two different body ideals: an athletic/sport ideal and a social/everyday ideal. Depending on the degree to which the psychological and aesthetic requirements of the particular sport contrast with mainstream culture’s ideals regarding female beauty and physique, female athletes may experience differing body dissatisfaction depending on their context at a particular moment [34]. Social physique anxiety and preoccupation with weight and shape are known to be greater for females during puberty, particularly in the early and late stages of puberty [35], and are related to increased body dissatisfaction and risk of developing an eating disorder [35]. There is evidence that young female athletes of pubertal age experience body image concerns at least equal to those that non-athlete females of pubertal age experience [36]. In one study, body image in a sample of swimmers and dancers aged 14 to 18 longitudinally and cross-sectionally predicted eating attitudes; these attitudes were consistent with a similarly aged non-athlete female sample [36].

### 3.3. Disordered Eating and Energy Deficiency

Along with body image, Martinsen and Sundgot-Borgen (2013) found a higher prevalence of eating disorders in elite adolescent female athletes (14.0%) compared to non-athlete female controls (5.1%) and male adolescent athletes (3.2%) [16]. Female athletes are known to present with distinct disordered eating and body image preoccupations [37]. Risk varies depending on the sport [38], with athletes in lean sports at greatest risk [37]. The higher prevalence in eating disorders in elite female adolescent athletes relative to other groups makes this population particularly vulnerable to the development of Relative Energy Deficiency Syndrome (RED-S) [39,40], which the International Olympic Committee (IOC; 2014) describes as energy deficiency relative to the balance between dietary energy intake and energy expenditure. RED-S affects many aspects of physiological functioning including overall health and athletic performance (issues with metabolic rate, menstrual function, bone health, immunity, protein synthesis, cardiovascular health) [41]. Unaddressed, RED-S may result in deepening physiological (decreased muscle strength, glycogen stores, aerobic and anaerobic performance, coordination, increased risk of injury) and psychological (impaired judgement, increased irritability, anxiety, and depressive symptoms, decreased concentration) health and performance in young female athletes [41]. Low energy availability and RED-S sequelae have been associated with both health and performance decrements in female adolescent and young adult athletes [40].

### 3.4. Fatigue and Poor Sleep

Female athletes with and without RED-S are at risk of fatigue and poor sleep. Poor sleep quality and short sleep duration in student-athletes is associated with mental health concerns [42], with poor sleep quality being shown to worsen mental health symptoms [43]. Further, chronic lack of sleep among young athletes is associated with greater risk for sport and musculoskeletal injuries [44]. A significant relationship between fatigue and psychological stress has also been demonstrated in female athletes [45]. With respect to the relationship between sleep and resilience, one study found female athletes had poorer sleep quality than males, and that resilience sub-components of social resources and structured style were positively associated with sleep quality, while worry and perceived stress were negatively associated [46].

### 3.5. Mental Distress

Resilience in athletes has been negatively associated with mental distress [47]. Female athletes may experience significant stressors and psychological challenges that, instead of enhancing resilience, can actually inhibit their athletic and personal potential. It is well known that females experience higher levels of internalizing disorders relative to men, who demonstrate higher levels of externalizing disorders [48]. For instance, females are nearly twice as likely to be diagnosed with depression than males [49] and are also more likely to experience anxiety [50]. Likewise, female athletes experience higher rates of anxiety and depression than male counterparts without a significant correlation with age [51]. Female athletes with anxiety symptoms are 1.9 times more likely to sustain injury than those without [52]. Athletes recovering from injuries are more likely to experience psychological distress during their recovery and subsequent treatment. Nearly 80% of surveyed athletes in one study reported psychological issues due to their injuries [52].

### 3.6. Minoritized Identities

Resilience is a particularly salient construct for female athletes with minoritized identities and experiences. Individuals from minority groups, including those from under resourced communities, have less access to quality athletic education and participation opportunities, as well as less access to health information and the resources needed to apply that information to support their health and athletic resilience. Additionally, minority and concealment stress in youth is well documented [53], and patterns of adversity and health outcomes are unequal. For example, racial and ethnic minority youth are observed to be at greater risk of experiencing higher levels of adversity and poor health outcomes compared to their white counterparts [54].

In addition to racial and ethnic minority athletes, LGBTQ+ individuals suffer from higher rates of mental health issues, like depression, in comparison to straight peers [55]. LGB youth are more likely to believe sports participation will include entering into an unwelcoming and unsafe environment, and consequently not profit from the physical and psychological health benefits [40]. In college, LGB athletes deal with fear of peer rejection, being “outed” and a lack of institutional support [56]. Lesbian female athletes experience homophobia through silence around lesbianism and through internalized stereotypes about lesbians, which leads these athletes to further internalize their oppression [57].

In addition to the salience of resilience for those with racial, ethic, and LGBTQ+ minoritized identities, it is also a key concept for young female athletes with disabilities. One study observed that athletes with disabilities had significantly lower mean scores on measures of resilience when compared to the mean scores reported by other studies using able-bodied populations. In this study, measures of resilience scores differed depending on the athlete’s particular disability, with resilience scores being lowest for individuals who had cerebral palsy, then intermediately low for individuals who had amputations and polio, and, finally, the highest (only moderate) scores for individuals who had spinal cord injuries and myelomeningocele [58].

### 3.7. Injuries

The relationship between stress and injury is bidirectional, making resilience a key component to both injury prevention and recovery among young female athletes. Challenges emerging from sport, life, and health events may prompt or exacerbate injury. For example, athletic burnout has been shown to be strongly negatively associated with resilience [59], with burnout frequently leading to injury [60]. In the other direction, becoming injured may trigger and/or exacerbate challenges to one’s sport performance, life, and/or health, making the concept of resilience particularly relevant. One study of athletes showed a significant positive relationship between anxiety and resilience capacity; however, an injury experience can impair resilience in injured athletes compared to those who are not injured, suggesting the potential negative impact of the stressful experience of an injury [61]. The psychological response to injury can prompt and/or expose underlying mental health issues including depression, anxiety, disordered eating, and substance use [62]. Resilience is an important asset when recovering from injury. In a study of elite female rhythmic gymnasts, Codonhato et al. (2018) found that resilience, particularly when bolstered by social support, optimized the process of injury recovery [63].

Although injuries can adversely impact an athlete’s health and wellness, these adverse experiences may also be viewed as a possible catalyst for new growth. For example, rehabilitation from injury has been associated with specific resilience factors. Johnson et al. (2016) studied resilience factors in the context of female soccer players’ rehabilitation after first-time anterior cruciate ligament injury and reconstruction and found that players who had the best injury outcomes had strong communication and relationships with significant others, held deep belief in value and effectiveness of their actions, and had the ability to set realistic goals [64]. One qualitative study of injured athletes found specific internal factors of personality, coping styles, knowledge, prior experience, perceived social support, external factors of cultural scripts, physical resources, time, and received social support as key components for personal growth following injury [65].

## 4. Psychological Resilience Benefits for Young Female Athletes

The ability to overcome adversity is crucial to any athlete, and psychological resilience has consistently been shown to benefit athletes [66,67,68]. When young female athletes can positively adapt to the stressors presented to them within a sport context, they have a better ability to respond to setbacks, obstacles, and failures [66] and transform adversity into an opportunity for personal growth [69]. Resilience is positively associated with athletic achievement [50,70] and psychological well-being [68]. Regardless of stress conditions, resilient athletes are less prone to burnout [71]. Resiliency has also been shown to enable athletes to turn adversity, such as sport-related injuries and mental health struggles, into personal growth [72]. Determining psychosocial and physiological predictors of resilience among young female athletes and helping them to develop skills to enhance resilience in the face of adversity may be key determinants of their athletic success and personal growth, which has the potential to carry over into their lives beyond sport. To determine these predictors, it is important to understand key individual and environmental contributors to resilience.

## 5. Factors that Enhance Psychological Resilience

### 5.1. Adversity and Positive Adaptation

Adversity, a crucial factor in the study of psychological resilience, has been defined as any experience of negative life circumstances associated with difficulties in adjustment as well as maladjustment [17,73]. Experiencing adversity and stress are necessary components in the development of resilience, as they allow one to overcome, learn, and grow. However, extreme stress can overwhelm the capacity for positive adaptation. Researchers studying concepts of resilience, adversity, and positive adaptation among young female athletes must consider a variety of adverse experiences associated with maladjustment in sport and/or non-sport domains of an athlete’s life. Adversity for young female athletes may include past or present negative life circumstances in sport and/or non-sport contexts. Stressors in the sport psychology literature are differentiated in terms of frequency (rare vs. common occurrence), intensity (high vs. low demand), and duration (chronic vs. acute) [11]. Ongoing daily stressors and hassles found specifically within the athletic experience include demands deriving from a competitive environment and athletic teams and institutions [21], including stress in athletic preparation, injury, expectations set by others (e.g., coaches, family member), concerns about self-presentation, and rivalry [11].

Positive adaptation is considered both a process (adaptation) and an outcome (positive) [74]. Mahoney and Bergman (2002) define positive adaptation as “the developmental processes by which individuals attain unusually favorable adjustment patterns, given their background and available resources” (p. 195) [75]. Close concepts to positive adaptation include “stress related growth” or “posttraumatic growth”, and “adversarial growth” [76], which assumes that one is not only “bouncing back” from and/or positively adapting to adversity but actually growing beyond where one would have been if one had not experienced that adversity [67,77,78]. A systematic review of studies on this process found that the conceptualization of growth following adversity is a process relying on internal mechanisms (i.e., mental toughness; [79] acceptance; [80] optimism; [81] and external; [80] having a cultural script involving a “quest narrative” [65,82]) as well as intrapersonal (i.e., deeper understanding of self and improved problem solving), [79] interpersonal (i.e., greater empathy), [83] being able to speak out and disclose, [82] and physical indicators (i.e., superior sport performance, [80] improved health behaviors and knowledge [65]).

### 5.2. Stress Coping Ability

Resilience has also been measured as a constellation of individual-level “stress coping” [19] and “protective” traits that include personality, cognitive, affective, and behavioral characteristics [21]. It has been proposed that individuals who possesses high standards, tenacity, trust in one’s instincts, the ability to tolerate negative affect, the ability to adapt to changing circumstances, and acceptance of oneself and one’s life situation are more likely to positively adapt to stress and adversity [19]. Feelings of personal competence, [19,22] engagement in self-leadership, [78] social skills, [22,84], and the ability to engage in behaviors that promote health and well-being have also been highlighted as predictive of positive stress coping [85]. Additionally, “hardiness” [83] or “personal hardiness” [86], as defined as an individual’s “courage and motivation to face stressors accurately (rather than to deny or catastrophize them)” [87], has been considered as a pathway to resilience by way of an individual approaching, rather than avoiding, existing stressors and engaging in problem-solving and seeking out encouragement and assistance from others. Being resilient requires the capacity to approach rather than avoid behavior [88] and to fully acknowledge the reality of stressful events, rather than avoid or deny their existence [89].

### 5.3. Supportive Athletic Environments

Strengths-based ecological approaches to the developmental of resilience, which emphasize promoting an athlete’s potential rather than preventing problems, have shown promise in enhancing resilience in young athletes [90]. Positive youth development (PYD), a multisystemic resiliency intervention targeting an increase in social connection, supportive parenting, and mentorship, is one such approach that has been applied to youth athlete populations with success [90].

Supportive coaching practices also play a critical role in fostering and maintaining resilience among athletes through their daily interactions [91]. One qualitative study found that coaches could be helpful in supporting resilience by fostering motivation, mental preparation, and promoting life balance for the athlete prior to a stressor and by evaluating setbacks, promoting a positive mindset, and implementing lessons for the athlete after a stressful event or experience [91]. These pre- and post-stressor coach supports were identified as more effective when these supportive actions were tailored to the athlete’s individual needs and also when the relationship between the coach and athlete was stronger. Studies have also examined athletes’ parent relationships, coach relationships, social connectivity, social support, and the relation to resilient-salient constructs such as well-being or performance. For example, coaches may assess their athletes for qualities of mental toughness such as challenge, control, commitment. and confidence and assist them in developing these traits, where they are needed [92].

## 6. Trainings and Interventions That Foster Mental Skills for Psychological Resilience

The goal of mental skills training is not only to improve athlete performance [93] but also to teach confidence, adaptability, and resilience. When athletes are better equipped to face adversity in sport, they are more likely to experience increased performance capabilities [94]. Psychological resilience training helps individuals to accurately perceive and then positively evaluate and interpret the pressure they encounter, together with their own resources, thoughts, and emotions [21]. Enhancing female athlete resilience leads to positive adaptation by building self-esteem, which has been implicated in better adjustment and stress coping [95]. Developing positive adaptation and self-esteem involves practicing acceptance and non-judgment about any negative thoughts so that they can begin, when they are ready, to positively adapt how they respond to such thoughts and beliefs [96].

Mindfulness and psychological skills-based techniques have been used as means to promote athletes’ mental toughness and improved performance outcomes [94,97,98,99]. Training in mindfulness has been shown to improve stress coping among female athletes [100]. Mindfulness practice increases non-judgmental awareness of the present [101] and is an effective skill in non-sport related arenas [97]. Mental skills training educates athletes on how to improve strategies such as imagery, self-talk, goal setting, and arousal regulation [93,102]. While these interventions may vary somewhat in terms of approach, there is evidence that both tactics positively impact athlete performance [94,97,98,99]. In addition, the consistent provision of motivational feedback is important to encourage and inform athletes about what has been and is effective in developing resilience [21].

A few formal psychological interventions to enhance psychological resilience also exist. One resilience training protocol for male and female adult athletes (i.e., Schinke and Peterson, 2002; Schinke and Jerome, 2002) focuses on psychological resilience just in the context of athletics, with the goal of improving or maintaining athletic performance, rather than a focus on a broader aim for psychological resilience encompassing athletic as well as other domains of functioning. Rooted in cognitive-behavioral theory and technique, the protocol targets improving performance by teaching athletes three cognitive skills, termed “general optimism” skills. The protocol has athletes evaluate one’s assumptions and develop an understanding of the chain of events that leads to the construction of those assumptions. It also teaches athletes to dispute one’s cognitive distortions. Finally, it offers a skill that helps athletes to de-catastrophize automatic thoughts of feared outcomes of a poor performance [103]. Other potential interventions that may facilitate resilience in the young female athlete are programs that target the parent–athlete relationship [104] and teammate social support [105], individual athlete experiential avoidance [106], and athlete-specific eating disorder prevention programs [107]. Resilience in young female athletes can be learned and enhanced, and these interventions can be successful at helping athletes maximize their potential.

## 7. Conclusions

In a world where the athletic participation of young females is increasing, distinct challenges such as gender and minority-based inequities, injury experiences, sexualization of female athletes in the media, tension experienced when the sport vs. the non-sport contexts hold discrepant body ideals, and chronic stress, fatigue, and mental distress highlight the importance of psychological resilience. Young female athletes may present with specific vulnerabilities including disordered eating, body dissatisfaction, anxiety, and depression, among others. These challenges warrant a closer look at how to develop and enhance resilience, the positive adaptation to adversity and stress coping ability, in this population. Further study of individual level characteristics and behaviors predictive of resilience would enable the development of targeted interventions to mitigate the effects of sport-related stressors and support the health, wellness, development, athletic, and other life aspirations of young female athletes.

## Data Availability

Data sharing not applicable.
